# Special Issue Recurrent Pregnancy Loss: Etiology, Diagnosis, and Therapy

**DOI:** 10.3390/jcm10215040

**Published:** 2021-10-28

**Authors:** Ole Bjarne Christiansen

**Affiliations:** Department of Obstetrics and Gynecology, Aalborg University Hospital, 9000 Aalborg, Denmark; olbc@rn.dk

The definition of recurrent pregnancy losses (RPL) varies between guidelines from different national and international scientific societies, but overall, a history of two or more (or alternatively, three or more) confirmed pregnancy losses is required for the diagnosis. Depending on the definition, 0.5% to 2.5% of all women suffer from RPL.

Most clinicians and researchers in the field of RPL recognize that the cause of the condition is multifactorial, with factors such as repeated fetal chromosome anomalies, the presence of antiphospholipid antibodies and clinical thyroid disease being recognized as risk factors/causes of RPL. Conversely, there is limited consensus about the importance of other factors such as maternal natural killer (NK) cells, heritable thrombophilia factors, cervical insufficiency and immunity against male-specific (HY) antigens. The controversies about which risk factors to screen for and which treatments should be used are highlighted in the review by Vomstein et al. [1].

Several papers in this Special Issue highlight and try to clarify the controversial issues in the area of RPL. Strobel et al. [2] report that the NK cell number and NK cytotoxicity in peripheral blood are higher in patients with primary RPL compared to secondary RPL. This is an important new finding which may explain controversies in previous studies of NK cells in RPL patients where samples taken from primary RPL patients are typically compared with samples from parous controls. If a previous birth really affects the number of NK cells permanently, as suggested in the study by Strobel et al. [2], only RPL patients and controls with similar parity should be compared in future studies of NK cells.

Guidelines agree that RPL patients should be screened for the anti-phospholipid (aPL) antibodies (lupus anticoagulant, anti-cardiolipin antibodies and β2-glycoprotein-I antibodies), whereas screening for other aPL antibodies such as antiphosphatidyl (aPS)/prothrombin (PT) antibodies is not recommended. The study by Pleguezuelo et al. [3] seems to indicate that aPS/PT antibodies may have a stronger association to RPL than the traditional aPL antibodies, with limited overlap between them. The results of the study call for more studies to clarify the importance of the non-traditional aPL antibodies.

There has been a growing focus on the role of peripheral blood extracellular vesicles in many late pregnancy complications, such as preeclampsia and gestational diabetes, but they have not previously been investigated in RPL. The placenta is a source of many kinds of extracellular vesicles during pregnancy, and the composition of these biomarkers during pregnancy may reflect the growth and “health status” of the trophoblast tissue, which may predict later pregnancy complications. In the study by Rajaratnam et al. [4], extracellular vesicles were measured before and several times during early pregnancy in a cohort of women with RPL. It was found that the levels of one type of extracellular vesicle with the surface marker CD9 (a general marker for extracellular microvesicles) were lower before pregnancy and increased more rapidly during early pregnancy in RPL patients who went on to miscarry again compared with those who gave birth. This calls for further studies on extracellular vesicles as disease markers in RPL. The study by Rajaratnam et al. [4] also found that infusions with intravenous immunoglobulin profoundly increased the levels of almost all kinds of extracellular vesicles in RPL patients, which may be one of the mechanisms through which intravenous immunoglobulin acts in the potential prevention of RPL.

A subset of RPL patients experience second trimester loss or preterm birth under a clinical picture of painless cervical dilatation or rupture of the membranes, traditionally called cervical insufficiency. Vaginal cerclage often fails in these cases, but a cohort study by Chung et al. [5], where 21.1% of the included patients had suffered three or more previous pregnancy losses, suggest that modified laparoscopic cervico-isthmic cerclage can increase the fetal survival rate. However, randomized trials are still needed to document the real benefits of this procedure. In the review by Wierzchowska-Opoka et al. [6], an overview is given of the procedures, outcomes and risk factors for failure after the application of emergency cerclage. This is, again, an area with no randomized controlled trials, although these would be difficult to carry out.

Several studies, especially from Danish research groups, have reported that a previous birth of a boy predisposes patients to subsequent RPL compared with a previous birth of a girl, and there are some indications that immunity against male-specific HY antigens is responsible for this clinical observation. The family-based study by Noergaard-Pedersen et al. [7] suggests that not only the previous birth of a boy, but also a history of an older brother can predispose to secondary RPL: the explanation for this observation may be the persistence of microchimaeric cells transferred through the placenta of the common mother from the older brother to the younger sister. This is the first (although indirect) evidence that microchimeric cells may play a role in the pathogenesis of RPL, although more studies are needed to confirm this.

During recent years, several studies have reported an increased incidence of obstetric and perinatal complications in women with a diagnosis of RPL. Most studies have been small, and there is still no strong consensus that RPL women are a high-risk population for adverse obstetrical and perinatal outcome. The large cohort study by Ticconi et al. [8] reports an increased incidence of intrauterine fetal death, preterm birth, fetal growth restriction preeclampsia, and placental abruption in RPL patients compared with a non-RPL control group. In the register-based study by Roepke et al. [9] based on all RPL patients in Sweden and a randomly selected register-based control group, these observations are confirmed. The risks of stillbirth, preterm birth, being small for gestational birth, preeclampsia, and placental abruption were all increased compared with controls with adjusted odds ratios between 1.45 and 2.47. There is now such overwhelming documentation that RPL patients are a high-risk population for obstetrical and perinatal complications that closer surveillance of the patients in late pregnancy must be introduced in clinical practice if not already done.

The study by Shiina [10] falls outside the subject area of the above-mentioned studies since it does not deal with risk factors for RPL or treatment of miscarriage, but rather reviews the knowledge about a rare neo-vascular lesion that can develop after delivery or miscarriage. The significance of this rare lesion is as yet uncertain.

Overall, the articles in this Special Issue of the *Journal of Clinical Medicine* should provide the reader with an overview of highlights from the very active research going on in this important area.
